# Changes in perceived psychosocial working conditions after psychotherapeutic consultation at work – exploratory post-hoc analyses from a randomized controlled trial

**DOI:** 10.1186/s12889-026-29388-5

**Published:** 2026-09-21

**Authors:** Jeannette Weber, Nicole Rosalinde Hander, Peter Angerer, Nadine Mulfinger, Ute Beate Schröder, Marieke Hansmann, Regina Herold, Christoph Kröger, Harald Gündel, Eva Rothermund-Nassir, Meike Heming

**Affiliations:** 1 Institute of Occupational, Social, and Environmental Medicine, Centre for Health and Society, Medical Faculty and University Hospital Düsseldorf, Heinrich Heine University Düsseldorf, Moorenstraße 5, Düsseldorf, 40225 Germany; 2 Department of Psychosomatic Medicine and Psychotherapy, Ulm University Medical Center, Ulm, Germany; 3 Department Psychiatry II, Section of Health Economics and Psychiatric Services Research, Ulm University & BKH Günzburg, Günzburg, Germany; 4 Division “Work and Health”, Federal Institute for Occupational Safety and Health, Berlin, Germany; 5 Institute of Psychology, University of Hildesheim, Hildesheim, Germany; 6 Department of Psychosomatic Medicine and Psychotherapy, University Hospital of Erlangen, Friedrich-Alexander University Erlangen-Nürnberg (FAU), Erlangen, Germany

**Keywords:** Psychotherapy, Prevention, Mental Health, Occupational Health, Job Demands-Resources model

## Abstract

**Background:**

Psychotherapeutic consultation at work (PT-A) is a low-threshold intervention providing early work-focused treatment to employees with symptoms of common mental disorders (CMD). This post-hoc analysis explores whether PT-A, compared with routine care, is associated with perceived reductions in job demands (i.e., quantitative and emotional demands, dissolution between work and private life) and improvements in job resources (i.e., decision authority, development opportunities, social support).

**Methods:**

The exploratory post-hoc analysis used data from a multicenter randomized controlled trial in Germany. Five-hundred-fifty employees with a contract of ≥ 15 h/week and symptoms or a diagnosis of CMD were randomized 1:1 to the intervention or control group. After one participant withdrew consent, 279 participants remained in the intervention and 270 in the control group. Both groups received basic clinical assessment. Up to 16 work-focused PT-A sessions were offered to the intervention group over nine months, including work-related assessments, work-focused cognitive behavioral therapy and, if needed, support during return-to-work. Perceived job demands and resources were measured before randomization and 24 months later and were not pre-specified as trial outcomes in the study protocol and trial registration. Multiple linear regression analyses examined group differences in perceived working conditions at follow-up, controlling for covariates at baseline (i.e., age, gender, education, occupation, study centre, depressive symptoms, sickness absence days).

**Results:**

Data from 231 participants were available for analysis (intervention: n = 137; control: *n* = 94; mean age: 46 years; 55% female). Participation in PT-A was associated with lower perceived emotional demands (b = 6.61, SE = 2.52, *p* = 0.009, adjusted *p* = .072) and dissolution (b = 5.05, SE = 2.52, *p* = 0.047, adjusted *p* = .164) at follow-up, but results did not remain statistically significant after Benjamini–Hochberg correction for multiple testing. No group differences were observed in perceived quantitative demands and job resources.

**Conclusions:**

Results may indicate that participation in PT-A is associated with long-term reductions in perceived emotional demands and dissolution, but not with improvement in perceived job resources. Confirmative research is needed given the exploratory study design and because findings did not remain statistically significant after adjustment for multiple testing.

**Trial registration:**

The study was registered on 1 March 2021 in the German Clinical Trials Register under the project-ID DRKS00023049 (https://drks.de/search/de/trial/DRKS00023049).

**Supplementary Information:**

The online version contains supplementary material available at https://doi.org/10.1186/s12889-026-29388-5.

## Background

Common mental disorders (CMD) are one of the main causes for sickness absence [1] and early retirement [2] in Germany. At the same time, working conditions influence mental health [3], a relationship being conceptualized within various work stress models, including the job demands-resources (JD-R) model [4–7]. According to the JD-R model, high levels of job demands lead to strain, whereas job resources foster work engagement and buffer negative effects of excessive job demands [4, 5]. A large range of prospective studies support the JD-R model by providing substantial evidence that job demands (e.g. quantitative and emotional demands) and a lack of job resources (e.g. decision authority, social support) increase the risk of mental disorders [3]. Likewise, high job demands and low levels of job resources were described as barriers for successful return-to-work (RTW) after being on sick-leave due to CMD [8]. Given this close relationship between work and mental health, the workplace represents an important setting for both the prevention and treatment of CMD. It not only constitutes a modifiable source of psychosocial risk and protective factors but also provides access to a large proportion of the adult population.

Psychotherapeutic consultation at work (PT-A) has therefore been developed as a low threshold intervention targeting especially employees at risk or in early disease phases of CMD [9–11]. PT-A and similar predecessor models aim to early identify and support employees with CMD symptoms and to bridge waiting times for routine mental health care [12, 13]. It is characterized by its proximity to the workplace, combines diagnostics with short-term work-focused psychotherapeutic treatment, but also assists employees upon RTW [9–11]. It thereby offers the opportunity to incorporate work-focused contents into treatment of CMD. While work-related assessments identify work stressors and resources, subsequent cognitive behavioral therapy (CBT)-related components include psychoeducation, problem solving, social and emotional skills training, and stress management [9, 10, 14, 15].

Those intervention components specifically aim to increase individuals’ competences to deal with work-related problems, demands and lack of work-related resources and to establish good social relationships [14]. PT-A might thereby facilitate a re-appraisal of psychosocial working conditions through changes in cognitions, behaviors and emotional states. More specifically, cognitive restructuring, a core CBT technique, may enable employees to identify and modify maladaptive interpretations of their work situation with more balanced and realistic appraisals [16–18]. This may reduce perceived job demands and increase awareness of job resources, even without objective changes in the work environment. Resource-oriented reflection may further enhance awareness of existing job resources including opportunities for decision-making, personal development or social support [14]. Development of problem-solving strategies and social and emotional competences may enable employees to express their needs more effectively, proactively shape their work environment, cope more effectively with emotional and quantitative job demands [14, 19, 20] and – as shown by previous research [21, 22]—may also improve perceived opportunities for decision authority or social support. PT-A may further reinforce these processes by increasing work-related self-efficacy [9, 23] and thus employees’ confidence in successfully managing their work tasks [24, 25]. In addition, PT-A related reductions in CMD symptoms [9] may improve emotion regulation and decrease cognitive biases in the processing of negative information [26, 27], potentially resulting in a more favorable perception of working conditions. This is in accordance to previous research suggesting a link between depressive symptoms and worse appraisal of job control [28]. Finally, work-related psychotherapy may promote stricter boundary-setting between work and private life [29].

Previous intervention studies provide preliminary support for an association between work-focused psychotherapy and perception of working conditions. For example, a randomized controlled trial (RCT) showed that interpersonal psychotherapy targeting psychosocial problems at work improves effort-reward imbalance [30]. Another controlled trial testing a work-focused CBT group intervention further showed that it reduced social work stressors and increased perceived control at work [22]. By contrast, a problem solving and CBT-based intervention that was not reported to be specifically work-focused reduced symptoms of depression, but showed no associations with perceived working conditions at follow-up [31]. Further small-scaled RCTs tested work-focused CBT among teachers and nurses and found that it was effective in reducing subjective general stress, work stress and role workload one to three months later [32, 33]. Those previous studies suggest that work-focused psychotherapy is effective in reducing work stress either as an early preventive measure [32, 33] or as tertiary prevention [22, 30]. They have, however, mainly investigated short-term effects one to three months after participating in the intervention and used well-defined occupational groups with a small sample size. Whether work-focused psychotherapeutic interventions are associated with sustained changes in employees’ perception of specific working conditions in heterogeneous working populations over longer follow-up periods remains largely unexplored.

One example for an elaborate concept of PT-A was developed and tested for effectiveness in the friaa study (German: Frühe Intervention am Arbeitsplatz; English: Early Intervention at the workplace). After a basic clinical diagnostic session, it offered up to 16 sessions of work-focused psychotherapeutic consultation with five sessions being reserved for psychotherapeutic support during RTW [10]. Within this multi-center RCT in Germany, 550 employees with (symptoms of) CMD were randomized into an intervention group (IG) receiving at least four sessions of PT-A and a control group (CG). The CG generally represents routine care with the exception that they participated in the first session of PT-A including a diagnostic clinical assessment and recommendations for routine care and also received one follow-up call. The study could not find evidence for the superiority of PT-A compared to routine care regarding reduction of days of sickness absence, but it did demonstrate superiority regarding improvement of CMD symptoms and work-related self-efficacy [9]. The friaa study provides the opportunity to explore whether participation in PT-A is also associated with long-term changes in the perception of psychosocial working conditions, which is highly important from a sustainability aspect since they predict mental well-being [3] and successful RTW [8]. This study therefore comprises an exploratory post-hoc analysis to explore whether employees in the IG perceived less job demands (i.e. quantitative demands, emotional demands, dissolution between private- and work-life) and more job resources (i.e. decision authority, development opportunities, social support) after a follow-up of 24 months than employees of the CG.

## Materials and methods

### Study design

The present study reports exploratory post-hoc analyses based on data from the previously conducted multicenter RCT, called friaa study, which had the main aim to test the effectiveness of PT-A regarding reduction of days of sickness absence compared to routine care. Participants were randomized into an IG and CG with both groups receiving one session of clinical diagnostic assessment. The IG additionally received up to 16 sessions of work-related psychotherapeutic intervention. Baseline data (T0) was obtained before randomization and participants were followed-up nine (T1) and 15 (T2) months after the clinical diagnostic assessment. A third follow-up took place 24 months (T3) after the intervention start to explore potential long-term effects of PT-A including changes in the perception of psychosocial working conditions. This study will use T0 and T3 data. The design, conduct and reporting of the study was discussed with a scientific advisory board. A protocol of the RCT was published before study start [10] and the study was registered on 1 March 2021 in the German Clinical Trials Register (DRKS) under the project ID DRKS00023049 (https://drks.de/search/de/trial/DRKS00023049). Results regarding days of sickness absence as the main outcome and information on harms were published elsewhere [9]. The analyses of the present paper including T3 and perception of psychosocial working conditions as additional outcomes were not pre-planned in the original study protocol or trial registration. T3 was planned after the start of the RCT, but before analysis of T1 and T2 data.

### Study participants

Participants were recruited around five study centers in Germany, located in Ulm, Erlangen, Düsseldorf, Hildesheim and Teltow from 09/2021 until 01/2023 and were blinded for study hypotheses. They were recruited by collaboration with 60 small, middle and large-sized companies, eleven inter-company services for occupational health and social counselling and through advertisements in newspapers and social media [34]. Participants received a gift card of 20€ after completion of the questionnaires at T0, T1 and T2 to increase motivation for further participation. All participants gave their written informed consent to participate in the study.

Inclusion criteria were current employment (≥ 15 h/week), age between 18 and 65 years and sufficient knowledge of the German language. A diagnosis of CMD following the International Statistical Classification of Diseases and Related Health Problems (ICD-10 with F-diagnoses F32-34, F40-45, F48.0, F51 including depressive, anxiety, stress-related and somatoform as well as non-organic sleep disorders; [35]) or symptoms of mental or psychosomatic disorders according to the Global Assessment of Functioning Scale (GAF score < 81; [36, 37]) were needed to participate in the study. Exclusion criteria were unique or main diagnoses of mental and behavioral disorders due to psychoactive substance use, schizophrenia, psychosis and organic psychiatric disorders according to ICD-10 (F-diagnoses F00-F29; [35]), because the intervention was not indicated for those diagnoses. Patients having other severe somatic health conditions such as cancer were excluded from the study due to expectable difficulties in attending PT-A. Persons already receiving psychotherapeutic treatment were excluded due to concomitant care and persons currently applying for early retirement were excluded due to competing goals. Diagnoses were made by assessing the medical and psychosomatic history and by conducting the Mini-International Neuropsychiatric Interview (M.I.N.I; [38]) by the study therapists. Persons with diverse gender were excluded within the analyses given their small number in the study (*n* = 1).

### Randomization

Participants were randomized to an IG and CG with 1:1 allocation using block randomization with variable block sizes of four, six and eight. As described elsewhere [9], randomization was stratified by days of sickness absence (< 21 days vs. ≥ 21 days within the previous six months) and severity of depression (Patient Health Questionnaire-9 (PHQ-9; [39, 40]), dichotomized into a sum score of < 11 vs. ≥ 11) to achieve a balanced allocation of participants with different levels of prior sickness absence and depressive symptoms between the IG and CG. This approach was chosen because baseline sickness absence is a well-established predictor of subsequent sickness absence [41], which was the main outcome of the original study [9]. Baseline severity of depression was chosen as another stratification factor due to evidence that it affects treatment response [42, 43]. In addition, randomization was stratified by study center to account for regional variation in population density and healthcare utilization [44], while also ensuring that study centers treated a comparable number of participants. A centralized web-based tool (www.randomizer.at) was used for randomization.

### Intervention

The manualized intervention called friaa comprised three modules according to an adapted version of a previous manual [14] and is described in more detail within the study protocol [10]. It comprised one basic clinical diagnostic and one work-related assessment with one session each (module 1), work-related psychotherapeutic consultation with up to ten sessions (module 2) and, if needed, psychotherapeutic consultation during RTW with up to five sessions (module 3). The CG only received the basic clinical diagnostic assessment of the first module, guidance on further procedures in routine care and a follow-up call by the psychotherapist three months later. This procedure was chosen due to ethical reasons. Aside from this, the CG represents routine care as no other treatment was delivered to the CG by the study team. Utilization of routine care was left to participants' discretion and was not controlled by the study. The IG received the full first module and at least two sessions within the second module according to patients’ needs. Participants could attend all sessions within a period of up to nine months. The basic clinical diagnostic session was scheduled for 100 min and all other sessions for 50 min. Licensed psychological or medical psychotherapists or therapists under postgraduate psychotherapeutic training, who were trained on the intervention and supervised by therapeutic and scientific staff of the study, conducted the intervention.

The work-related assessment included a detailed analysis of variables such as work experience, job requirements and tasks, person-job fit, social conflicts and working conditions (first module). Work-related psychotherapeutic consultation in the second module comprised short-term CBT or cognitive-behavioral prevention, conditional on the severity of symptoms. This included development of a personalized stress model considering workplace factors, imaginal exercise to explore work-related self-efficacy and problem solving during the first two sessions of the second module. During the imaginal exercise, participants rehearsed a typical workday, identified work-related resources and challenges, and imaginatively practiced coping with difficult situations to enhance perceived occupational self-efficacy [45]. Further optional therapy components such as relaxation and coping techniques, development of emotional and social competences or exposure therapy could be applied according to patients’ needs during subsequent sessions. The third module comprised psychotherapeutic support in planning (e.g., work analysis, formulation of a reintegration plan) and evaluating RTW.

Therapists received a comprehensive treatment manual, instructional videos, training, and supervision to support standardized intervention delivery. As described elsewhere [9], treatment adherence was evaluated by rating video recordings of 182 therapy sessions, indicating a high level of adherence to the treatment protocol.

Details on criteria for discontinuation and modifications of the intervention are outlined in the study protocol [10].

### Measures

Baseline and follow-up data were obtained by online questionnaires. Pen and paper questionnaires were only used in case of technical problems or in case of difficult access to the participants in follow-up assessments.

#### Perception of psychosocial working conditions

The validated third German version of the Copenhagen Psychosocial Questionnaire (COPSOQ) was used to measure perception of psychosocial working conditions at T0 and T3 with the following sub-scales [46, 47]: quantitative job demands by five items (example: “Do you have to do overtime?”), emotional demands by two items (example: “Is your work emotionally demanding?”), dissolution between work and private life (example: “I take care of work-related tasks outside of my working time as well”), decision authority by two items (example: “Can you influence the amount of work assigned to you?”), development opportunities by three items (example: “Is your work varied?”), social support by four items (example: “How often do you get help and support from you colleagues, if needed?”; [46]). Five-point rating scales were used for all items, ranging from 0 = ”never or hardly never” to 100 = ”always” or from 0 = “to a very small extent” to 100 = “to a very large extent” according to previous validation and reliability studies [46, 47]. Mean values per sub-scale were calculated when participants answered at least half of the items. Higher scores indicate more job demands and more job resources. Reliability was good for quantitative demands (Cronbach’s alpha (CA) = 0.80), emotional demands (CA = 0.84, Spearman-Brown coefficient (SC) = 0.84) and social support (CA = 0.80), acceptable for development opportunities (CA = 0.75) and questionable for dissolution (CA = 0.63, SC = 0.63) and decision authority (CA = 0.62, SC = 0.62).

#### Covariates

Age, gender (female, male), education (highest education: no secondary school, lower secondary school, intermediate secondary school, grammar school, university), occupation (blue- or white-collar worker), depressive symptoms (dichotomized sum score of the PHQ-9: < 11 vs. ≥ 11; [39, 40]), days of sickness absence (< 21 days vs. ≥ 21 days within the previous six months) and study centre were used as co-variables. They were chosen as covariates to enhance precision of effect estimates due to the association of sociodemographic variables with perceived working conditions [48, 49] or because variables were used as stratifying factors during randomization (i.e., depressive symptoms, days of sickness absence, study centre; [50]). Except for education, which was measured at T2, data on co-variables were collected at T0.

#### Additional variables

Data on current employment status, subjective work ability and somatic symptoms were collected at all time points and used as additional variables to predict missing data during multiple imputation. Current employment status was measured by asking which labor situation is most accurate right now (dichotomized into current employment: fully employed, partly employed, traineeship vs. no current employment: unemployed, student, homemaker, (early) retirement, voluntary work). Subjective work ability was measured by the first item of the Work Ability Index (WAI; [51, 52]) and somatic symptoms by the validated Somatic Symptoms Scale-8 (SSS; [53]).

### Statistical analyses

A drop-out analysis compared the characteristics of participants who dropped out of the study with those who completed the survey at T3 using chi-squared tests and t-tests for all dependent variables and covariates. Univariate analyses examined changes in perceived working condition from T0 to T3 within each group (IG and CG) by using paired t-tests.

Multiple linear regression analyses were performed to examine differences in the IG and CG on perceived working conditions at T3 while controlling for perceived working conditions at baseline and covariates. The IG was used as the reference group (IG = 0, CG = 1) within the analyses. Multicollinearity was assessed using the variance inflation factor (VIF), which was below 1.5 for all models. Linearity was confirmed graphically with component-plus-residual plots. Normality of residuals was confirmed graphically using histograms and normal Q-Q plots. Homoscedasticity was evaluated graphically using a residuals-versus-fitted plots and formally tested using the studentized Breusch-Pagan test. Heteroscedasticity was detected in the models predicting perceived emotional demands, dissolution and social support. For these models, regression coefficients were reported with heteroscedasticity-robust standard errors (HC3).

Multiple linear regression analyses were repeated as a sensitivity analysis after multiple imputation of missing data due to loss-to-follow-up at T3 and assuming missing-at-random. The R package “mice” was used to perform multiple imputation of missing data for all variables at T2 and T3 [54]. 100 completed datasets were generated by predictive mean matching using a predictor matrix being automatically generated by considering group (IG vs. CG), all dependent variables at T0 and T3, all co-variates and the PHQ-9 total score, SSS total score, WAI and current employment status at T0, T2 and T3. Only group was always included as a predictor. Results of multiple linear regression analyses of the imputed datasets were then pooled using Rubin’s rule [55]. Subgroup analyses were conducted by excluding participants who did not work at T3 by using the non-imputed dataset.

All analyses were performed in R-4.1.3. Statistical significance was assumed at a *p*-level < 0.05. As a sensitivity analysis, *p*-values were adjusted for multiple testing using the Benjamini–Hochberg procedure with a false-discovery rate of 0.05 [56]. Sample size calculations are outlined in the study protocol [10].

## Results

### Flow of participants

**Fig. 1:**
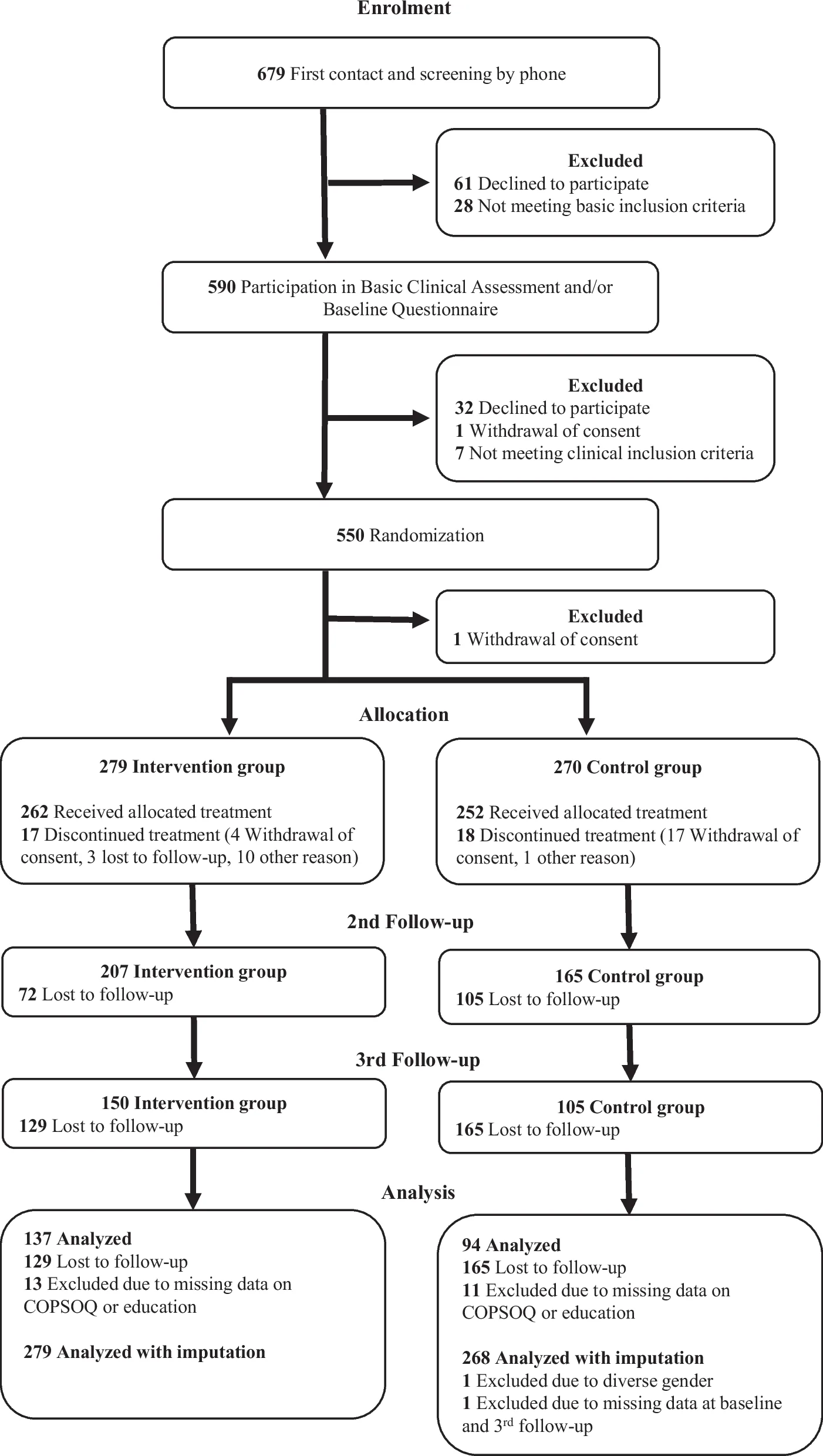
Flow diagram, adapted from the CONSORT statement [43]

A flow-chart on participation is given in Fig. 1. After randomization, one person withdrew their consent for participation and data usage. This resulted in 549 participants being allocated to the IG or CG. From 372 persons participating at T2, 255 persons could also be followed-up at T3. However, not all persons being followed-up at T3 also provided data on the COPSOQ or on education, resulting in 231 participants for the complete-case-analyses with a loss-to-follow-up of 58%. For analyses with multiple imputation of missing data, one participant was excluded due to diverse gender and one participant due to missing data at baseline. This resulted in 547 participants for the imputed analyses and included all participants which were also used for the complete-case analyses.

### Drop-out analyses

Table 1 describes baseline characteristics of drop-outs (not participating at T3) and participants providing data at T3. No differences were observed regarding gender, education, age, perception of emotional demands, dissolution and social support. Participants providing data at T3 reported lower depressive symptoms and more perceived decision authority and development opportunities than drop-outs. A similar pattern was observed for depressive symptoms and perception of development opportunities when stratifying drop-out analyses for the IG and CG (Supplemental Material, Table S1). Perception of decision authority only differed in the IG when comparing participants providing data at T3 (M = 42.88, SD = 22.63) with drop-outs (M = 35.92, SD = 24.41; t = −2.47, df = 276.6, *p* < 0.05). Those analyses suggest that differential loss-to-follow-up between the IG and CG has occurred for perception of decision authority but not for the other baseline characteristics. To analyze whether loss-to-follow-up is related to changes in depressive symptoms, those analyses were repeated investigating differences between participants providing data at T3 (*n* = 231) and drop-outs participating at T2 but not T3 (*n* = 136). Those analyses suggest that loss-to-follow-up is marginally related to changes in depressive symptoms (Drop-Outs: M(Δ_T2-T0_) = −4.00 (SD = 5.92); Participants at T3: M(Δ_T2-T0_) = −5.13 (SD = 5.38); t = 1.83 (df = 262.2), *p* = 0.068), but in a similar pattern within both groups (Supplemental Material, Table S2).

**Table 1:** Drop-out analysis comparing baseline characteristics of Drop-Outs and Participants at T3

	Drop-outs (*n* = 316)		Participants T3 (*n* = 231)		Test statistic (ꭓ^2^ or t-test)^f^			
	n (%)	M (SD)	n (%)	M (SD)	ꭓ^2^	t	df	*p*
Female gender	168 (53)		133 (58)		0.88		1	.349
Highest education^a^					3.05		3	.385
- No secondary school	0 (0)		0 (0)					
- Lower secondary school^b^	12 (10)		19 (8)					
- Intermediate secondary school^c^	35 (30)		56 (24)					
- Grammar school^d^	25 (21)		45 (20)					
- University^e^	45 (39)		111 (48)					
Age		45.89 (11.57)		45.97 (10.29)		−0.09	542.4	.928
PHQ (sum-score)		13.35 (5.03)		12.30 (5.20)		2.38	486.5	**.018**
Quantitative Demands		54.40 (20.93)		56.40 (22.07)		−1.07	480.24	.285
Emotional Demands		48.44 (32.72)		49.03 (32.41)		−0.21	498.29	.836
Dissolution		33.40 (27.13)		34.31 (26.53)		−0.90	501.59	.697
Decision authority		38.37 (23.72)		42.64 (21.96)		−2.17	515.73	**.030**
Development Opportunities		60.81 (21.63)		66.77 (20.93)		−3.25	504.96	**.001**
Social Support		58.16 (23.16)		60.62 (22.06)		−1.26	508.32	.208

*Abbreviations*: *df* Degrees of freedom, *M* Mean, *n* Number, *p p*-value, *PHQ* Patient Health Questionnaire, *SD* Standard deviation, *T3* Third follow-up

Statistical significance at *p* < = .05 shown in bold

^a^Education was surveyed at T2. Drop-outs for this variable thus represent individuals participating at T2 but not T3

^b^school-leaving certificate after nine years of school (German: Hauptschule)

^c^school-leaving certificate after ten years of school (German: Realschule)

^d^school-leaving certificate after 12–13 years of school (German: Gymnasium)

^e^General and applied-science university

^f^compares baseline characteristics between Drop-Outs and Participants T3

### Descriptive results

There were slightly more female than male participants at T3. About half of the participants had a university degree and mean age was 45.97 years (see Table 1).

Table 2 provides descriptive statistics for perceived working conditions at T0 and T3 and explores within-group changes from T0 to T3 by paired t-tests. These analyses are descriptive only and are not intended to address the main research question. Average scores of perceived quantitative demands decreased in both groups. Average scores of perceived emotional demands and dissolution decreased and average scores of perceived social support increased from T0 to T3 in the IG. No marked changes were observed for the perception of decision authority and development opportunities.

**Table 2:** Descriptive analyses of perceived working conditions at T0 and T3

	Intervention group						Control group					
	T0	T3	Paired t-test^a^				T0	T3	Paired t-test^b^			
	M (SD)	M(SD)	n	t	df	*p*	M (SD)	M(SD)	n	t	df	*p*
Quantitative demands	56.90 (21.31)	49.45 (20.69)	137	4.57	136	** <.001**	55.68 (23.23)	51.88 (21.67)	94	2.26	93	**.026**
Emotional demands	50.36 (31.06)	45.62 (31.01)	137	2.40	136	**.018**	47.07 (34.36)	49.34 (29.18)	94	−1.11	93	.272
Dissolution	35.49 (26.96)	26.55 (23.16)	137	4.82	136	** <.001**	32.58 (25.94)	30.19 (22.41)	94	1.047	93	.298
Decision authority	42.88 (22.63)	45.89 (21.28)	137	−1.83	136	.070	42.29 (21.07)	45.48 (22.14)	94	−1.529	93	.130
Development opportunities	66.18 (20.38)	65.81 (18.32)	136	0.12	135	.907	67.64 (21.79)	68.93 (21.56)	94	−0.707	93	.481
Social support	58.29 (21.50)	63.29 (21.19)	135	−3.01	134	**.003**	64.03 (22.53)	66.80 (22.95)	94	−1.185	93	.239

^a^compares perceived working conditions at T0 and T3 within the intervention group

^b^compares perceived working conditions at T0 and T3 within the control group

*Abbreviations*: *df* Degrees of freedom, *M* Mean, *n* Number, *p p*-value, *SD* Standard deviation, *T0* Baseline, *T3* Third follow-up

Statistical significance at *p* < = .05 shown in bold

### Main results

The primary analyses—comparing the IG and CG—were based on multiple linear regression models predicting perceived working conditions at T3 while adjusting for baseline values and covariates. Their results are given in Tables 3 and 4. Significant differences between the IG and CG were only found for the perception of emotional demands and dissolution, indicating that perceived emotional demands were 6.61 points and perceived dissolution was 5.05 points higher at T3 in the CG compared to the IG. However, those finding did not remain robust when using Benjamini–Hochberg adjusted *p*-values (adj. *p* = 0.072 for emotional demands and adj. *p* = 0.164 for dissolution). No significant differences between the IG and CG were observed with regard to perceived quantitative demands, decision authority, development opportunities and social support. Similar results were obtained under multiple imputation of missing data (Supplemental Material, table S3 and table S4).

**Table 3:** Multiple linear regression models predicting perceived job demands at T3, without imputation

	Quantitative demands (*n* = 231)				Emotional demands (*n* = 231)				Dissolution (*n* = 231)			
	b	ß	SE	*p*	b	ß	SE	*p*	b	ß	SE	*p*
Outcome variable at T0	0.58	0.61	0.05	** <.001**	0.68	0.72	0.04	** <.001**	0.52	0.61	0.05	** <.001**
Age	0.06	0.03	0.11	.592	0.31	0.11	0.13	**.021**	0.19	0.08	0.13	.147
Gender (ref. male)	4.71	0.11	2.27	**.039**	3.93	0.06	2.64	.138	0.84	0.02	2.76	.760
Education	−0.46	−0.02	1.30	.723	3.33	0.11	1.40	**.018**	0.42	0.02	1.55	.788
Occupation (ref. blue collar)	4.92	0.09	3.24	0.130	−1.84	−0.02	4.02	0.65	−0.68	−0.01	3.50	0.846
PHQ at T0 (ref. sum-score < 11)	−0.85	−0.02	2.24	.706	−1.41	−0.02	2.89	.627	1.48	0.03	2.52	.557
Days of sickness absence at T0 (ref. < 21 days)	−3.55	−0.08	2.43	0.145	−3.76	−0.06	3.17	0.236	−3.30	−0.07	2.87	0.252
Group (ref. IG)	3.36	0.08	2.15	.120	6.61	0.11	2.52	**.009**	5.05	0.11	2.52	**.047**
Model fit	Adj. R^2^ = 0.46, F(12,218) = 15.38, *p* <.001				Adj. R^2^ = 0.59, F(12,218) = 28.51, *p* <.001				Adj. R^2^ = 0.363, F(12,218) = 11.91, *p* <.001			

All model were adjusted for study centre

Statistical significance at *p* < = .05 shown in bold

*Abbreviations*: *adj.* Adjusted, *b* Not standardized regression estimate, *ß* Standardized regression estimate, *IG* Intervention group, *n* Number, *p p*-value, *PHQ* Patient Health Questionnaire, *ref.* Reference group (scored as 0), *SE* Standard Error, *T0* Baseline, T3 Third follow-up

**Table 4:** Multiple linear regression models predicting perceived job resources at T3, without imputation

	Decision authority (*n* = 231)				Development opportunities (*n* = 230)				Social support (*n* = 229)			
	b	ß	St.E	*p*	b	ß	St.E	*p*	b	ß	St.E	*p*
Outcome variable at T0	0.56	0.57	0.06	** <.001**	0.54	0.58	0.05	** <.001**	0.48	0.48	0.06	** <.001**
Age	−0.23	−0.11	0.12	.065	−0.13	−0.07	0.11	.263	−0.24	−0.11	0.13	.072
Gender (ref. male)	−0.89	−0.02	2.42	.713	0.79	0.02	2.20	.721	3.91	0.09	2.70	.150
Education	1.06	0.05	1.39	.445	−0.71	−0.10	1.28	.582	0.80	0.04	1.66	.631
Occupation (ref. blue collar)	1.04	0.02	3.44	.763	4.53	0.09	3.10	.146	−6.97	−0.13	3.72	.062
PHQ at T0 (ref. sum-score < 11)	−2.98	−0.07	2.41	.218	−4.06	−0.10	2.21	.067	−1.32	−0.03	2.72	.628
Days of sickness absence at T0 (ref. < 21 days)	1.03	0.02	2.62	.694	−3.69	−0.09	2.36	.120	−3.95	−0.08	3.07	.199
Group (ref. IG)	0.11	0.00	2.32	.962	2.02	0.05	2.10	.339	0.41	0.00	2.67	.878
Model fit	Adj. R^2^ = 0.37, F(12,218) = 12.14, *p* <.001				Adj. R^2^ = 0.38, F(12,217) = 12.64, *p* <.001				Adj. R^2^ = 0.29, F(12,216) = 8.83, *p* <.001			

All model were adjusted for study centre

Statistical significance at *p* < = .05 shown in bold

*Abbreviations*: *adj.* Adjusted, *b* Not standardized regression estimate, *ß* Standardized regression estimate, *IG* Intervention group, *n* Number, *p p*-value, *PHQ* Patient Health Questionnaire, *ref.* Reference group (scored as 0), *SE* Standard Error, *ref.* Reference group, *T0* Baseline, *T3* Third follow-up

Subgroup analyses of multiple linear regression analyses predicting perceived working conditions at T3 were performed excluding participants who indicated that they did not work at T3 due to unemployment (*n* = 5), (re-)education (*n* = 1), occupational disability (*n* = 2) or other reasons (*n* = 2). Results of those analyses are shown in the supplemental material (table S5). They were similar to the above-mentioned analyses with all participants, except that the association between the intervention and perceived emotional demands increased in strength and remained significant when using Benjamini–Hochberg adjusted *p*-values (b = 7.31, ß = 0.12, SE = 2.62, *p* = 0.006, adj.*p* = 0.048).

## Discussion

This RCT may suggest that participation in PT-A is associated with reductions in perceived emotional demands and dissolution between work and private life among employees with CMD symptoms, but that it is not associated with perceived quantitative demands or perceived job resources. These results underline previous evidence that work-focused CBT can lead to a decrease in job stress [30, 32, 33] and suggests that those effects are enduring over a period of two years. However, these findings should be interpreted cautiously and require confirmation in future studies due to the exploratory study design and the risk that significant findings were only found due to multiple testing. The risk that significant findings were only found due to multiple testing is especially high for perceived dissolution as shown by the insignificant Benjamini–Hochberg adjusted p-value. The finding for perceived emotional demands appears to be slightly more robust, as the Benjamini–Hochberg adjusted p-value remained close to statistical significance and the effect remained statistically significant in the sensitivity analysis excluding participants who reported that they did not work at T3. Our findings further support results of a previous study on an early intervention comprising problem solving and CBT for employees being at risk of sick leave due to depressive disorders, which also found no differences between the IG and CG regarding perception of quantitative job demands, decision authority and social support at follow-up [31].

Regarding practical significance of the findings, the adjusted mean difference of 6.61 points between the IG and CG for perception of emotional demands corresponds to approximately one quarter of a response category on the original five-point scale (25 points per response category) of the COPSOQ. Regarding perceived dissolution, the adjusted mean difference of 5.05 points between the IG and CG corresponds to approximately one fifth of a response category. Even though those differences seem small, one should keep in mind that the intervention was tailored to individual needs. Participants did not uniformly receive components potentially addressing emotional demands and dissolution between work and private-life. Therefore, the observed overall association may underestimate the potential impact of specific intervention components in participants for whom they are indicated. To the best of our knowledge, no established threshold values for practically or clinically meaningful changes exist for COPSOQ scales. However, the strength of effects within our study are comparable to those of mindfulness-based interventions. For example, comparable reductions in general perceived job demands and dissolution were found in a sample of healthy managers after participating in a mindfulness-based intervention [57].

The observed reductions in the perception of emotional demands and dissolution may reflect a combination of cognitive change and behavioral mechanisms rather than actual changes in the work environment. Because potential mechanisms of change were not examined by this study, they should be regarded as hypotheses for future research. It is conceivable that psychoeducation and development of an individual strain model [10, 14] increased participants’ awareness of emotional job demands and blurring boundaries between work and private life, while cognitive restructuring changed participants’ perspective how to interpret and evaluate work-related stressors [16–18]. The intervention might have further improved emotional regulation, setting boundaries, and reattributing responsibility for interpersonal demands at work. Furthermore, the intervention explicitly targets increased work-related self-efficacy [23], which may have boosted participants’ confidence in managing emotionally demanding situations.

Likewise, behavioral strategies such as problem solving, relaxation and stress coping techniques [10, 14, 19] may have helped participants to better cope with emotionally demanding situations, eventually leading to the feeling that emotional job demands have decreased. The intervention may also have encouraged patients to establish clearer boundaries between work and their private lives. This assumption is supported by qualitative findings showing that employees on RTW after CMD-related sick leave placed greater meaning to their private life and reduced dissolution of work and private life [58].

Last but not least, the intervention-related reduction of CMD symptoms [9] may also have contributed to a more positive perception of emotional job demands and dissolution between work and private life. Depression and anxiety are associated with impaired emotion regulation and cognitive biases in the processing of negative information [26, 27]. Consequently, reductions in these symptoms may result in a more favorable appraisal of working conditions. As described as a downward-spiral process of negative emotion, perceived stress may further increase symptoms of depression and anxiety, which again reinforce the perception of stress [59]. Such reciprocal relationships have also specifically been described for job strain and depressive symptoms [29, 60]. However, the present study cannot disentangle whether improvements in mental health changed participants' perceptions of their job demands, whether changes in perceived job demands contributed to symptom improvement, or whether both outcomes resulted from shared intervention mechanisms.

In contrast, no associations were observed between the intervention and perceived job resources. One possible explanation is that the intervention primarily targeted work-related problems rather than building resources at work. In addition, job resources might be more strongly tied to the organization and a closer contact to the employer or its’ occupational health professionals might be needed during the intervention to facilitate adaptations of working conditions. Furthermore, only a minority of participants received the RTW module that encouraged communication with employers about working conditions. These explanations remain speculative and should be examined in future studies.

### Limitations

That the T3 assessment and perception of working conditions as outcomes were not pre-specified in the original study protocol or trial registration is one of the major limitations of the present study. Consequently, findings should be interpreted as exploratory and hypothesis-generating. Nevertheless, the analyses were theoretically informed and provide important insights into potential longer-term intervention effects on the perception of psychosocial working conditions, an outcome that is rarely assessed when studying the effectiveness of PT-A and related concepts.

Another major limitation of the study is the high loss-to-follow-up from T0 to T3. For most baseline variables, no differences were observed between participants of T3 and drop-outs. However, lower baseline levels of decision authority were related to loss-to-follow-up, especially within the IG. This differential drop-out may have resulted in an underestimation of the association between PT-A and decision authority if participants with a greater need for improvement were more likely to drop out. Conversely, the association between PT-A and perception of emotional demands or dissolution may have been overestimated if participants with less perceived control over changing these working conditions were more likely to drop out. Loss-to-follow-up might have further led to an overestimation of the associations when individuals of the IG who felt that the intervention did not help dropped out or may led to an underestimation of the associations when individuals of the CG dropped out who did not improve. As a proxy to estimate the risk of this bias, we have investigated whether loss-to-follow-up is related to changes in depressive symptoms from T0 to T2, but no substantial differences were observed between the IG and CG. Taken together, several mechanisms may have resulted in selective loss-to-follow-up. Therefore, the direction and magnitude of any resulting selection bias cannot be determined with certainty and our results should be interpreted with caution. However, sensitivity analyses based on multiple imputation yielded comparable results, indicating that the main findings were reasonably robust to missing outcome data, although bias due to data missing not at random cannot be ruled out.

Furthermore, our results may not be generalizable to the general German workforce, since employees without migration background, from large-sized companies (see [9]) and with a university degree are overrepresented in our study sample.

Having only two measurements per participant also impeded use of multilevel analyses, which could have accommodated missing observations. Integrating not all scales in each follow-up assessment was decided to keep participants’ burden low regarding answering lengthy questionnaires. We have therefore assessed perception of psychosocial working conditions only at T3, because we assumed that cognitive change and coping mechanisms require some time to be implemented in working life to change the perception of working conditions.

As we compared a complex intervention with routine care, the observed associations cannot be attributed to specific intervention components. Furthermore, it cannot be determined whether they were attributable to the work-focused psychotherapeutic intervention or only to the improved access to psychotherapeutic care in general. In addition, participants in both groups may have received additional treatments as part of routine care during the long follow-up period. However, no differences in overall treatment costs under routine care were observed at T2 between the IG and CG [61], suggesting that differential treatment outside the trial is unlikely to have systematically biased the results.

The exclusive use of self-reported outcomes made it impossible to understand whether changes are related to a different appraisal or actual change in working conditions. Furthermore, internal reliability was questionable for the COPSOQ scales of dissolution and decision authority, potentially leading to an attenuation of their associations with the intervention. One should however keep in mind that internal reliability depends on the number of items [35]. Smaller reliability estimates can therefore be expected for both scales, consisting of only two items each.

### Implications

The results indicate that the intervention may be beneficial for employees with symptoms of CMD to reduce their perception of emotional job demands and dissolution. These results add to our previous findings that PT-A leads to alleviation of depression, anxiety and somatic symptoms [9]. PT-A thus represents a complementary targeted individual-level prevention tool to promote mental health at work in addition to prevention on the company level such as identification of adverse working conditions and establishing more universal measures to reduce them.

More in-depth research including qualitative approaches is also needed on the pathways on how this intervention might be related to the perception of working conditions and why no associations with perceived quantitative job demands and job resources were observed. Those results could also inform further development of intervention components to accomplish improvement of perceived job resources as well. This would be important, since improvement of perceived job resources might contribute to employees’ well-being by increasing work engagement and buffering negative effects of job demands according to the JD-R model [4]. In this sense, not only job demands but also decision authority, social support and job strain as a combination of high job demands and low decision authority have been related to burnout and depression [3]. In addition, future research might evaluate whether module 3, which encourages patients to discuss their working situation with their employer upon RTW, or a closer network between the intervention and occupational health professionals can support objective changes of working conditions, when appropriate.

## Conclusions

Findings of this study may suggest that PT-A offering work-focused CBT as a targeted, low-threshold prevention tool is associated with a reduction of perceived emotional job demands and possibly dissolution between work and private life. The intervention was however not associated with the perception of quantitative job demands and job resources. Given the exploratory nature of the analyses and multiple testing, the findings should be interpreted as hypothesis-generating and warrant confirmation in future studies.

## Supplementary Information

Supplementary Material 1: Table S1. Drop-Out Analysis comparing the intervention and control group regarding baseline characteristics of Drop-Outs and Participants at T3. Table S2. Drop-Out analysis comparing participants of T3 with drop-outs participating in T2 but not T3 regarding change of PHQ from T0 to T2. Table S3. Multiple linear regression models predicting perceived job demands at T3, with imputation (*n*=547). Table S4. Multiple linear regression models predicting perceived job resources at T3, with imputation (*n*=547). Table S5. Multiple linear regression models without imputation for the subgroup of participants working at T3.

## Data Availability

The data that support the findings of this study are not publicly available due to privacy reasons but are available from the corresponding authors upon reasonable request.

## Abbreviations

CBT: Cognitive behavioral therapy
CG: Control group
CMD: Common mental disorders
GAF: Global Assessment of Functioning Scale
ICD: International classification of disease
IG: Intervention group
PHQ-9: Patient Health Questionnaire 9
PT-A: Psychotherapeutic consultation at work
RCT: Randomized controlled trial
RTW: Return to work
T0: Baseline
T1: First follow-up
T2: Second follow-up
T3: Third follow-up
