# Supplementary material for: Changes in perceived psychosocial working conditions after psychotherapeutic consultation at work – exploratory post-hoc analyses from a randomized controlled trial
Source: BMC Public Health. 2026 Sep 21;26:2638. doi: 10.1186/s12889-026-29388-5 (PMC13595506; doi:10.1186/s12889-026-29388-5)
Supplement: Supplementary file 1 [file 12889_2026_29388_MOESM1_ESM.pdf]

Results of additional analyses

Changes in perceived psychosocial working conditions after psychotherapeutic consultation at work – Exploratory post-hoc analyses from a randomized controlled trial

Jeannette Weber[1*], Nicole Hander[2], Peter Angerer[1], Nadine Mulfinger[3], Ute Beate Schröder[4], Marieke Hansmann[5], Regina Herold[6], Christoph Kröger[5], Harald Gündel[2], Eva Rothermund-Nassir[2], Meike Heming[1]

[1] *Institute of Occupational, Social, and Environmental Medicine, Centre for Health and Society, Medical Faculty and University Hospital Düsseldorf, Heinrich Heine University Düsseldorf, Düsseldorf, Germany*

[2] *Department of Psychosomatic Medicine and Psychotherapy, Ulm University Medical Center, Ulm, Germany*

[3] *Department Psychiatry II, Section of Health Economics and Psychiatric Services Research, Ulm University & BKH Günzburg, Günzburg, Germany*

[4] *Division "Work and Health", Federal Institute for Occupational Safety and Health, Berlin, Germany*

[5] *Institute of Psychology, University of Hildesheim, Hildesheim, Germany*

[6] *Department of Psychosomatic Medicine and Psychotherapy, University Hospital of Erlangen, Friedrich-Alexander University Erlangen-Nürnberg (FAU), Erlangen, Germany*

* Institute of Occupational, Social, and Environmental Medicine, Centre for Health and Society, Medical Faculty and University Hospital Düsseldorf, Heinrich-Heine University Düsseldorf, Moorenstraße 5, 40225 Düsseldorf, Germany; Jeannette.Weber@uni-duesseldorf.de

Table S1. Drop-Out Analysis comparing the intervention and control group regarding baseline characteristics of Drop-Outs and Participants at T3

	**Drop-Outs**			**Participants T3**			**Drop-Outs vs. Participants T3**	
	**IG (n=142)**	**CG (n=174)**	**IG vs CG[6]**	**IG (n=137)**	**CG (n=94)**	**IG vs CG[7]**	**IG[8]**	**CG[9]**
	n (%)/ M(SD)	n (%)/ M(SD)	χ^2/t (df)	n (%)/ M(SD)	n (%)/ M(SD)	χ^2/t (df)	χ^2/t (df)	χ^2/t (df)
Gender (female)	77 (54)	91 (52)	0.052 (1)	82 (60)	51 (54)	0.50(1)	0.69 (1)	0.03 (1)
Highest education[1]			1.71 (3)			1.06(3)	5.12 (3)	0.10 (3)
- Lower secondary school[2]	7 (12)	5 (9)		12 (9)	7 (7)			
- Intermediate secondary school[3]	19 (33)	16 (27)		30 (22)	26 (28)			
- Grammar school[4]	13 (22)	12 (20)		27 (20)	18 (19)			
- University[5]	19 (33)	26 (44)		68 (50)	43 (46)			
Age	46.92 (10.93)	45.04 (12.03)	1.45 (310.4)	45.89 (10.11)	46.10 (10.60)	-0.15(193.8)	0.82 (276.5)	-0.74 (212.2)
PHQ	13.50 (5.08)	13.24 (5.01)	0.46 (299.6)	12.63 (5.21)	11.82 (5.17)	1.16 (201.1)	1.41 (276.0)	2.16 (185.6)
Quantitative Demands	54.43 (20.85)	54.38 (21.05)	0.02 (302.6)	56.90 (21.31)	55.68 (23.23)	0.41 (188.4)	-0.98 (276.1)	-0.45 (175.3)
Emotional Demands	47.41 (33.24)	49.28 (32.37)	-0.50 (294.2)	50.36 (13.06)	47.07 (34.36)	0.74 (186.4)	-0.76 (274.4)	0.51 (181.5)
Dissolution	33.45 (27.05)	33.38 (27.27)	0.03 (296.41)	35.49 (26.96)	32.58 (25.94)	0.83 (205.0)	-0.63 (274.0)	0.23 (199.7)
Decision authority	35.92 (24.41)	40.37 (23.02)	-1.66 (293.8)	42.88 (22.63)	42.29 (21.07)	0.20 (209.0)	**-2.47 (276.6)***	-0.69 (205.7)
Development Opportunities	60.56 (21.33)	61.02 (21.93)	-0.19 (304.5)	66.18 (20.38)	67.64 (21.79)	-0.51 (191.1)	**-2.25 (277.0)***	**-2.37 (191.8)***
Social Support	55.80 (23.84)	60.05 (22.49)	-1.61 (287.9)	58.29 (21.50)	64.03 (22.53)	-1.94 (193.8)	-0.91 (271.9)	-1.38 (190.4)

Notes: [1] Education was surveyed at second follow-up. Drop-outs thus represent individuals participating at T2 but not T3; [2] school-leaving certificate after nine years of school (German: Hauptschule)¸[3] school-leaving certificate after ten years of school (German: Realschule); [4] school-leaving certificate after 12-13 years of school (German: Gymnasium); [5] General and applied-science university; [6] compares Drop-Outs of the intervention and control group; [7] compares Participants at T3 of the intervention and control group; [8] compares Drop-Outs and Participants at T3 within the intervention group; [9] compares Drop-Outs and Participants at T3 within the control group; * for p-level < .05; Abbreviations: CG=control group, df=degrees of freedom, IG=intervention group, M = mean, n=number, p=p-value, PHQ = Patient Health Questionnaire, SD=standard deviation, T3=third follow-up

Table S2. Drop-Out analysis comparing participants of T3 with drop-outs participating in T2 but not T3 regarding change of PHQ from T0 to T2

	n	M (SD)	t (df), p value
Drop-Outs	136	-4.00 (5.92)	1.83 (262.2), .068
Participants T3	231	-5.13 (5.38)	
Drop-Outs of IG	69	-4.64 (6.03)	1.34 (125.4), .183
Participants T3 of IG	137	-5.80 (5.48)	
Drop-Outs of CG	67	-3.34 (5.76)	0.94 (131.5), .349
Participants T3 of CG	94	-4.17 (5.11)	

Notes: Abbreviations: CG=control group, df=degrees of freedom, IG=intervention group, M=mean, n=number, PHQ=Patient Health Questionnaire, SD=standard deviation, T0=baseline, T2=second follow-up, T3=third follow-up

Table S3. Multiple linear regression models predicting perceived job demands at T3, with imputation (n=547)

	**Quantitative demands**			**Emotional demands**			**Dissolution**		
	b	St.E.	p	b	St.E.	p	b	St.E.	p
Outcome variable at T0	0.55	0.05	**<.001**	0.69	0.04	**<.001**	0.51	0.05	**<.001**
Age	0.06	0.09	.498	0.11	0.11	.328	0.09	0.11	.414
Gender (ref. male)	4.55	1.88	**.016**	4.02	2.73	.144	0.73	2.11	.729
Education	-0.51	1.22	.677	2.57	1.53	.097	0.08	1.20	.948
Occupation (ref. blue collar)	5.66	3.05	.067	-1.14	3.70	.758	-1.11	3.68	.764
PHQ at T0 (ref. sum-score<11)	-2.16	2.18	.324	-3.91	2.73	.155	2.08	2.60	.425
Days of sickness absence at T0 (ref. <21 days)	-1.39	1.84	.451	-2.36	2.28	.302	-1.53	2.32	.509
Group (ref. IG)	3.70	1.98	.063	6.69	2.51	**.009**	5.19	2.61	**.049**

Notes: All model were adjusted for study centre; Abbreviations: b=not standardized regression estimate, IG=intervention group, n=number, p=p-value, PHQ=Patient Health Questionnaire, ref.=reference group (scored as 0), SE=Standard Error, T0=baseline, T3=third follow-up

Table S4. Multiple linear regression models predicting perceived job resources at T3, with imputation (n=547)

	**Decision authority**			**Development opportunities**			**Social support**		
	b	SE	p	b	SE	p	b	SE	p
Outcome variable at T0	0.55	0.05	**<.001**	0.55	0.05	**<.001**	0.48	0.05	**<.001**
Age	-0.16	0.11	.148	-0.12	0.10	.245	-0.25	0.11	**.031**
Gender (ref. male)	0.20	2.07	.923	-0.27	1.74	.875	1.07	2.08	.607
Education	1.64	1.50	.279	-0.17	1.21	.890	0.83	1.22	.499
Occupation (ref. blue collar)	-2.41	3.53	.496	1.76	2.97	.555	-6.20	3.24	.058
PHQ at T0 (ref. sum-score<11)	-2.40	2.26	.290	-3.17	2.02	.118	0.65	2.37	.783
Days of sickness absence at T0 (ref. <21 days)	-1.08	2.14	.614	-2.20	2.14	.306	-1.31	2.24	.558
Group (ref. IG)	-0.66	2.28	.772	2.05	1.92	.287	0.59	2.37	.803

Notes: All model were adjusted for study centre; Abbreviations: b=not standardized regression estimate, IG=intervention group, n=number, p=p-value, PHQ=Patient Health Questionnaire, ref.=reference group (scored as 0), SE=Standard Error; ref.=reference group, T0=baseline, T3=third follow-up

Table S5. Multiple linear regression models without imputation for the subgroup of participants working at T3

	**Quantitative demands (n=221)**			**Emotional demands (n=221)**			**Dissolution (n=221)**			**Decision authority (n=221)**			**Development opportunities (n=221)**			**Social support (n=220)**		
	b	St.E.	p	b	St.E.	p	b	St.E.	p	b	St.E.	p	b	St.E.	p	b	St.E.	p
Outcome variable at T0	0.565	0.055	**<.001**	0.676	0.037	**<.001**	0.521	0.051	**<.001**	0.555	0.058	**<.001**	0.537	0.056	**<.001**	0.461	0.064	**<.001**
Age	0.073	0.121	.547	0.303	0.142	**.034**	0.176	0.133	.188	-0.201	0.131	.127	-0.153	0.117	.192	-0.267	0.140	.058
Gender (ref. male)	4.516	2.380	.059	4.562	2.723	.093	0.828	2.774	.766	-1.362	2.537	.592	0.530	2.286	.817	3.496	2.777	.210
Education	-0.311	1.358	.819	3.284	1.469	**.026**	0.196	1.580	.902	0.921	1.454	.527	-0.796	1.331	.551	0.482	1.686	.775
Occupation (ref. blue collar)	4.648	3.381	.171	-2.068	4.149	.619	-2.078	3.601	.564	0.619	3.590	.863	5.466	3.213	0.518	-5.815	3.749	.122
PHQ at T0 (ref. sum-score<11)	-0.349	2.336	.881	-1.819	2.995	.544	1.363	2.469	.581	-3.222	2.508	.200	-3.860	2.286	**.093**	-1.110	2.775	.690
DOSA at T0 (ref. <21 days)	-3.280	2.575	.204	-4.619	3.352	.170	-5.027	2.778	.072	0.951	2.773	.732	-3.557	2.486	**.154**	-4.832	3.235	.137
Group (ref. IG)	3.228	2.240	.151	7.314	2.617	**.006**	4.984	2.492	**.047**	0.703	2.410	.771	1.403	2.169	.518	0.291	2.715	.915
Model fit	Adj. R^2=0.401, $F(12,208)$=13.29, p<.001			Adj. R^2=0.588, $F(12,208)$=27.25, p<.001			Adj. R^2=0.390, $F(12,208)$=12.74, p<.001			Adj. R^2=0.354, $F(12,208)$=11.05, p<.001			Adj. R^2=0.371, $F(12,208)$=11.81, p<.001			Adj. R^2=0.273, $F(12,207)$=7.86, p<.001		

Notes: All model were adjusted for study centre; Abbreviations: b=not standardized regression estimate, DOSA=Days of sickness absence, IG=intervention group, n=number, p=p-value, PHQ=Patient Health Questionnaire, ref.=reference group (scored as 0), SE=Standard Error, T0=baseline, T3=third follow-up
